# Genetic diversity of the Tibetan antelope (*Pantholops hodgsonii*) population of Ladakh, India, its relationship with other populations and conservation implications

**DOI:** 10.1186/s13104-016-2271-4

**Published:** 2016-10-21

**Authors:** Khursheed Ahmad, Ved P. Kumar, Bheem Dutt Joshi, Mohamed Raza, Parag Nigam, Anzara Anjum Khan, Surendra P. Goyal

**Affiliations:** 1 Centre for Mountain Wildlife Sciences, Faculty of Veterinary Sciences and Animal Husbandry, Sher-e-Kashmir University of Agricultural Sciences and Technology, Srinagar, Jammu and Kashmir 190006 India; 2 Wildlife Forensic and Conservation Genetics Cell, Wildlife Institute of India, Post Box #18, Chandrabani, Dehradun, Uttarakhand 248001 India; 3 Wildlife Health Managment, Wildlife Institute of India, Post Box #18, Chandrabani, Dehradun, Uttarakhand 248001 India

**Keywords:** mtDNA, Shahtoosh wool, Poaching and conservation, Tibetan antelope

## Abstract

**Background:**

The Tibetan antelope (*Pantholops hodgsonii*), or chiru, is an endangered antelope, distributed in China [Xinjiang, Xizang, Qinghai, Zhuolaihu Lake (Breeding habitat)], and India (Aksai Chin and Ladakh). There is a global demand for the species prized wool, which is used in weaving shahtoosh shawls. Over the years, the population of the Tibetan antelope has drastically declined from more than a million to a few thousand individuals, mainly due to poaching. Field studies undertaken in Ladakh, India also indicated winter migration of the population to Tibet. Migration between winter and calving habitats is well established to be female-biased across the Qinghai Tibetan Plateau (QTP). For effective conservation planning, genetic characterization is considered the best way to understand the likely impact of threats for ensuring the long-term viability of the population. In this regard, genetic characteristics of all Chinese populations are well-studied using mitochondrial and microsatellite markers, but information is lacking for the Indian population. Therefore, using the control region marker, we document for the first time the genetic variation of the Indian population of the Tibetan antelope, the extent of migration and its relationships with other populations of China.

**Results:**

The partial fragment of control region (259 bp) marker was successfully amplified in 30 Tibetan antelope samples that were collected from the Chang Chenmo Valley in eastern Ladakh, India. We also retrieved control region sequences (n = 88) available in the public domain from GenBank of different Chinese populations. Low levels of nucleotide (π; 0.004) and haplotype (*hd*; 0.543) diversity were observed in the Indian population when compared to Chinese populations (π = 0.01357–0.02048 and *hd* = 0.889–0.986). Commonly used indices (Tajima's *D* and Fu's *Fs*) were analyzed for inferring the demographic history of the Indian populations, and all values were negative indicating population expansion or demographic equilibrium, though nonsignificant. We observed five haplotypes in the Indian population, and these were not reported in previously studied populations of QTP. Bayesian-based phylogenetic analysis indicates the presence of four clades, however, the posterior probability support for three of these clades is weak (<0.5). Of these, the Indian population formed a distinct clade, whereas the Chinese populations exhibited shared haplotypes, and no geographic structure was observed. Median-joining network analysis was conducted for 46 haplotypes in the overall population, except the samples from India which showed a star-like topology. The Indian population is separated by one median vector from the Chinese population.

**Conclusions:**

The present study revealed the presence of different sub-clades in the Bayesian phylogenetic tree and five new haplotypes only in the Indian population or sampling location. Furthermore, in the phylogenetic tree, Indian haplotypes of Tibetan antelopes were clustered with the haplotype reported in the Chinese population of the Xinjiang region. Median-joining network analysis showed shared haplotypes pattern in all populations of QTP except the samples from India which showed new haplotypes. Given the presence of low nucleotide and haplotype diversity in eastern Ladakh populations and limited information available for populations of the western side in its range, we suggest to include genetic studies of Tibetan antelope populations around Aksai Chin (Fig. 1) under the proposed transboundary agenda between India and China and assess relationships with other populations. Such understanding would enable the planning of conservation strategies for ensuring long-term survival of westernmost populations in its range, and if required, it would establish connectivity with the other populations.

## Background

The Tibetan antelope (*Pantholops hodgsonii*), also known as chiru has very recently been reclassified on the International Union for Conservation of Nature (IUCN) Red list as near threatened due to the recovery of some populations [1]. The species is listed in Appendix 1 of the Convention On International Trade In Endangered Species (CITES) (1979) [2], and in Schedule I of the Indian Wildlife (Protection) Act 1972. It is a Class I protected species under the Law of the People’s Republic of China on the Protection of Wildlife (1989).

**Fig. 1:**
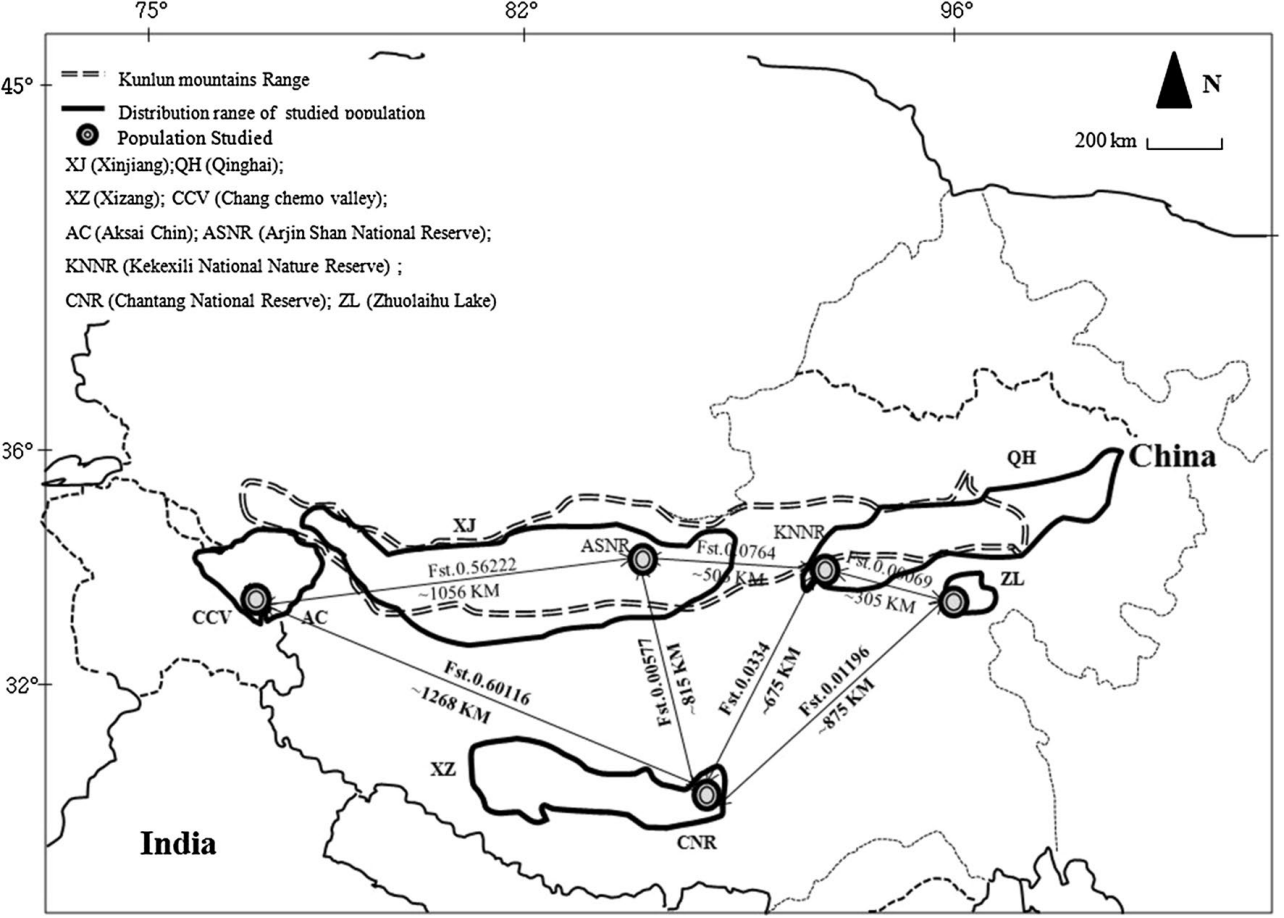
Current distribution of Tibetan antelope (*Pantholops hodgsonii*) in the Tibetan Plateau of the Chinese provinces of Tibet, Qinghai, Xinjiang and India

The Tibetan antelope is endemic to the Qinghai Tibetan Plateau (QTP) and occupies open-high elevation alpine and desert steppe habitats in the mountainous terrain with frequent occurrence at elevations between 3250 and 5500 m [3–6]. The present population occurs, almost exclusively, in about 800,000 km^2^ of the Chinese provinces of Xizang (XZ), Xinjiang (XJ), Qinghai (QH) [7–11], Zhuolaihu Lake (breeding habitat; BH), and in very small numbers in north-western India (Aksai Chin and Ladakh), mainly in summer [12–14] (Fig. 1). The Tibetan antelope has rarely been found in Nepal [15–18]. At the beginning of the last century, more than a million Tibetan antelopes were present, but more than 50 % of individuals have been reduced in just 20 years of the 20th century due to habitat fragmentation and increased human activity such as poaching, hunting and smuggling of product [5, 10, 11]. Approximately 75,000 Tibetan antelopes are now reported in their distributional range [19, 20]. Zhuolaihu Lake is known as one of the major breeding habitats (BH) or calving ground for Tibetan antelopes present in the Qinghai province where during the summer season, thousands of females migrate there from wintering habitats to breed [21]. It has been suggested that the migratory behavior exhibited by the Tibetan antelope promotes genetic exchange between different meta-populations and maintains all the populations together as one panmictic population [21].

In India, Tibetan antelopes are found in Daulat Beg Oldi (DBO) and the Chang Chenmo Valley (CCV) in north–east Ladakh (Fig. 1) [13, 14]. The average altitude of these areas is 4725–5500 m, and Tibetan antelopes are found between the altitudes of 4709–4964 m (mean 4797 ± 27.9 m). These areas are mostly surrounded by glaciers and rivers. Unequal individual numbers were sighted from the DBO: 230 individuals during 2005 and 45 individuals in 2006. In the CCV, a total of 55–60 individuals of Tibetan antelopes were reported during 2005. These two Indian populations (DBO and CCV) are isolated from one another due to mountains and river barriers. In DBO, both males and females have been reported [22, 23], whereas in CCV, only males have been seen [11]. Though migration from wintering to calving habitats is well-documented on QTP, little is known for such movements of the population of India. It has been reported that the male Tibetan antelope population in CCV in India is resident [23]. In contrast to these males, individual females travel distances of 300–400 km in late spring and early summer [24]. About 300 individuals seasonally migrate to India from the Chinese provinces of Tibet (Xinjiang area) [12], though it is not clear whether this population is truly migratory like the other populations of QTP [21, 25, 26] or is resident [22, 23].

Tibetan antelopes are poached for their wool, known as ‘shahtoosh,’ a layer of dense, fine wool on their skin that traps warm air, from which fine yarn is produced. This commands a high price [27, 28]. Shahtoosh is different from other wools as it can’t be sheared or combed because the fibres are very short and attached to the bases of guard hairs; in turn, it can only be obtained after sacrificing the animal. One individual yields about 125–150 g of shahtoosh, and weaving one shawl requires wool from four to five individuals [11]. It is estimated that every year, more than 20,000 individuals are poached for their fine wool to make shahtoosh shawls and scarves [29–31]. These garments can be sold for $1000–5000 USD for a single piece and sometimes as much as $15,000–17,600 USD depending on the length [31, 32]. It has been documented that when large numbers of animals are harvested or removed from the wild population due to poaching and other factors, the genetic diversity and population structure is severely affected by decreasing effective population size [33, 34].

A limited number of studies have been conducted so far on the genetic aspects of Tibetan antelope populations, and these studies have revealed that Tibetan antelopes have female-biased dispersal with a high mutation rate of mitochondrial DNA (mtDNA) [10, 19], which is a good marker for identifying female-based linkages between populations [35–38]. Hence, we hypothesized that the Tibetan antelope population of India is also genetically connected with the rest of the populations because of the observed migratory behavior on QTP. Therefore, we describe, for the first time, genetic variation in the hypervariable control region of the mtDNA genome and describe its relationships with other populations.

## Methods

### Sample collection

Four hair samples and 26 fecal samples were collected from different locations in Chang Chenmo Valley (Ladakh District), Jammu and Kashmir, India (IND 1–30) (Table 1) during field work. Collected hair samples were kept in an envelope and supplied with the sampling location and sample ID. The fecal pellets were collected and air dried to remove all moisture. The pellets were then kept in sterile vials containing silica gel and brought into the laboratory.

**Table 1:** Details of samples and mtDNA diversity (π and *hd*) of Tibetan antelope using control region (259 bp) marker from five locations

Population	n	Variable site	No. of haplotypes	Nucleotide diversity (*π*)	Haplotype diversity (*hd*)	Tajima's *D*	Fu’s *Fs*	Source*
IND	30	9	5	0.00458	0.543	−0.71389	−0.295	Present study
XJ	17	15	12	0.01357	0.956	−1.29957	−5.770	Ruan et al. [10]
XZ	18	19	11	0.01474	0.889	−1.45806	−3.542	Ruan et al. [10]
QH	18	19	9	0.02048	0.895	−0.5069	−0.376	Ruan et al. [10]
BH	35	21	17	0.01712	0.921	−0.70218	−5.533	Zhang et al. [19]
Overall	118	83	54	0.01810	0.927	−1.32871	−23.551	

* The analysis is only performed with the 259 bp

### DNA extraction and PCR amplification

Genomic DNA was extracted from hair samples using a hair extraction kit (Merck, GeNei™), and pellet genomic DNA was isolated using a commercially available kit (Qiagen DNeasy Stool Kit, Qiagen, Germany) according to the manufacturer’s instructions. To avoid contamination, each sample was processed separately using new sterile blade during DNA extraction, and negatives control were included to monitor contamination. Amplification of the hypervariable fragment of control region (259 bp) marker was amplified using an ABI 2720 thermocycler with a pair of primers [38]. The polymerase chain reaction (PCR) was conducted in 20 µl reaction volumes that contained 1× PCR buffer, 25 mM MgCl_2_, 10 mM dNTPs, 10 µM of each primer, 0.5 U Taq polymerase (MBI, Fermentas, USA), and approximate 20 ng of genomic DNA. To check for DNA contamination, a negative control was set up with a PCR master mix. The PCR cycling conditions were the following: initial denaturation (94 °C) for 5 min, followed by 40 cycles of denaturation (40 s at 94 °C), annealing (50 s at 56 °C), extension (40 s at 72 °C), and a final extension at 72 °C (10 min). Amplification of PCR was checked on gel electrophoresis using 2 % agarose gel.

### DNA sequencing and analysis

The PCR products were purified using ExoSAP (exonuclease I-shrimp alkaline phosphatase) and finally sequenced on an ABI 3130 genetic analyzer using a Big Dye Terminator v 3.1 Kit (Applied Biosystem, USA). Sequences of the control region (n = 88) of different Chinese populations of Tibetan antelope were retrieved from the work of Ruan et al. [10] and Zhang et al. [19] (Table 1). The goat (*Capra hircus)* was used as an outgroup in our study. The alignments of the control region sequences of Tibetan antelopes with the complete control region of goat (AF533441) were aligned using Clustal W as implemented in BioEdit version 7.0.9.0 [39]. The sequence divergence between haplotypes (H) was determined using a MEGA version 6.0 [40]. We calculated the haplotype (*hd*) and nucleotide diversity (π) using DnaSP version 3.5.1 [41] to check the level of genetic diversity in the Indian population and compared these diversity indices with other Chinese populations of Tibetan antelope. Neutrality tests were performed using the software Arlequin version 3.0 [42] and DnaSP, version 3.5.1 [41] as these values indicates recent population expansion or contraction. Phylogenetic tree using the Bayesian inferences (BI) were conducted in BEAST version 2.1.3 [43]. A median-joining haplotype network analysis was constructed using the Network version 4.5.1 (http://www.fluxusengineering.com).

Patterns of historical demography were inferred from the estimates of effective population size over time using the Bayesian skyline plot (BSP) implemented in BEAST version. 2.1.3. We used the substitution rate according to Zhang et al. [19] which was used for Tibetan antelopes (2 % per million year for the control region gene). This method estimates a posterior distribution of effective population sizes through time via MCMC procedures. The constant population size coalescent model was the basic assumption used for this approach. Together, among-site rate heterogeneity across all branches and a strict molecular clock were used for this calculation. Markov chains were run for 2.5 × 10^7^ generations and were sampled every 1000 generations with the first 2500 samples discarded as burn-in. Other parameters were set as default values, and results were visualized in TRACER version 1.6 [44].

## Results and discussion

The partial fragment of the control region (259 bp) was sequenced successfully for all 30 samples of the Indian population. Nine polymorphic and two singleton variable sites were found (Table 1) which defined a total five H. After combining the control region sequences of the four Chinese populations (n = 88) and the Indian population (n = 30), a total of 83 variable (polymorphic) sites were observed which constitutes a total of 46 H considering gaps or missing sites. The nucleotide frequencies were as follows: A-41.14 %, T-28.20 %, C-21.17 %, and G-9.49 %.

The overall *hd* and π within the Indian population were 0.543 and 0.00458. The combined analysis of the Indian and Chinese samples revealed that the overall haplotype and nucleotide diversity were 0.927 and 0.01810, respectively (Table 1). The values of nucleotide (0.00458) and haplotype diversity (0.543) indicates that the low diversity within the Indian population in comparison to the Chinese populations (π = 0.01357–0.02048; *hd* = 0.889–0.956). Additionally, these diversity indices were also lower when compared with the data set of Ruan et al. [10] (π 0.02178; *hd* 0.997) based on more than 1 kb pair (Table 1).

A comparison of diversity indices to other endangered populations of antelope and deer species indicate similar ranges of diversity viz. Roan antelope (*Hippotragus equinus*; π 0.000–0.030; *hd* 0.500–1.00) [45], Saiga antelope (*Saiga tatarica*; π 0.00478; *hd* 0.918) [46], and Kashmir red deer, (*Cervus elaphus hanglu;* π 0.008; *hd* 0.589) [47]. Observed low genetic diversity in Chiru is similar to the Kashmir red deer population which is documented to have undergone a major decline due to habitat degradation and anthropogenic pressure [48]. However, the nucleotide diversity observed in the Indian population of Tibetan antelopes is half of that observed in Kashmir red deer (0.008), which is the only surviving population of this species in the Indian subcontinent and has declined in its distribution range. Hence, red deer population of India has been suggested to be promoted in the endangered category [47]. Observed loss of genetic diversity of Indian population in comparison to Chinese populations of Tibetan antelopes requires immediate conservation attention.

**Fig. 2:**
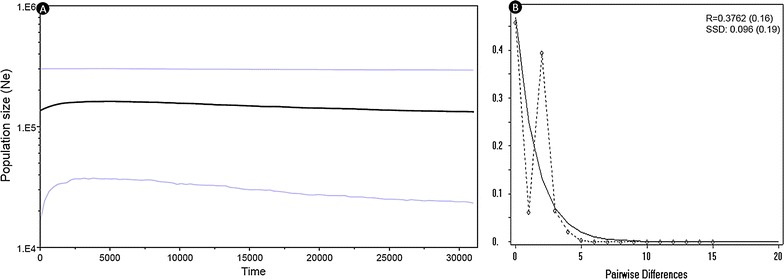
Bayesian skyline plot for Tibetan antelope of Indian population based on 259 bp control region gene (**A**) and mismatch distribution graph (**B**)

The sequence divergence between the different populations of Tibetan antelope was 0.014–0.028 %, whereas it is low (0.019) between the Indian and the Xinjiang populations in comparison to the other three Chinese populations (Table 2). The Indian population indicates high Fst (>0.50; p = 0.001) with other Chinese populations, whereas values ranged only between 0.00069 and 0.0764 in spite of large geographical distances among Chinese populations (305–815 km) (Fig. 1). Our analysis indicates that the Indian population has been isolated from the rest of the Chinese populations; however, the level of sequence divergence indicates that the Chinese population of Xinjiang is closer to the Indian population than the rest of the populations. Matel test analysis indicates isolation by distance (r = 0.6154; p = 0.90) in all the Tibetan antelope populations but p value is not significant. We performed Tajima’s *D* and Fu’s *Fs* tests for understanding demographic history in Indian and other Tibetan antelope populations (Table 1). Tajima’s *D* and Fu’s *F*s statistics values were negative, though they were non-significant in the Indian population. Similarly, negatives values of Tajima’s *D* and Fu’s *F*s were also reported in earlier studies on Tibetan antelopes [10, 19, 25]. Overall mismatch distribution pattern analysis (Fig. 2) of the Indian population showed weak multimodal; however, shape of patterns may be considered unimodal which suggests signs of population expansion. Likewise, published studies on QTP have also reported negative values in demographic history indices and a unimodal mismatch distribution pattern in overall populations except one as reported by Du et al. [25]. However, Zhang et al. [19] found the presence of weak multimodal mismatch patterns; yet, the shape was in agreement with the model of population growth. In addition, we also observed non-significant sum of square deviations and raggedness in all the populations, which indicates that data are a relatively good fit to a model of population expansion [49]. All these analyses, including ours, reveal historic population expansion, and suggests that colonization of the Indian population was part of this expansion. However, we observed relatively higher values of the raggedness index (though non-significant in the Indian population) which may suggest stable or bottlenecked populations, whereas these values in populations of QTP were low [19]. Bayesian skyline plot (BSP) analysis of the Indian population (Fig. 2) indicated the population remained constant for a long time, and there has been a recent decline ca. around 5 Kyr. Such analysis undertaken for the QTP population has highlighted constant population during early-mid Quaternary and expansion during mid Holocene. However, declines in population size were also observed around 5 Kyr [25]. These declines may have been due to anthropogenic activities and environmental changes [10, 19, 25].

**Table 2:** Estimates of sequence divergence over the sequence pairs between groups (lower diagonal) and pair wise population F_ST_ between groups (upper diagonal)

Population	IND	XJ	XZ	QH	BH
IND		0.56222	0.60116	0.57918	0.53813
XJ	0.019		0.00577	0.0764	0.01928
XZ	0.023	0.014		0.03341	0.01196
QH	0.028	0.019	0.019		0.00069
BH	0.023	0.015	0.016	0.019

**Fig. 3:**
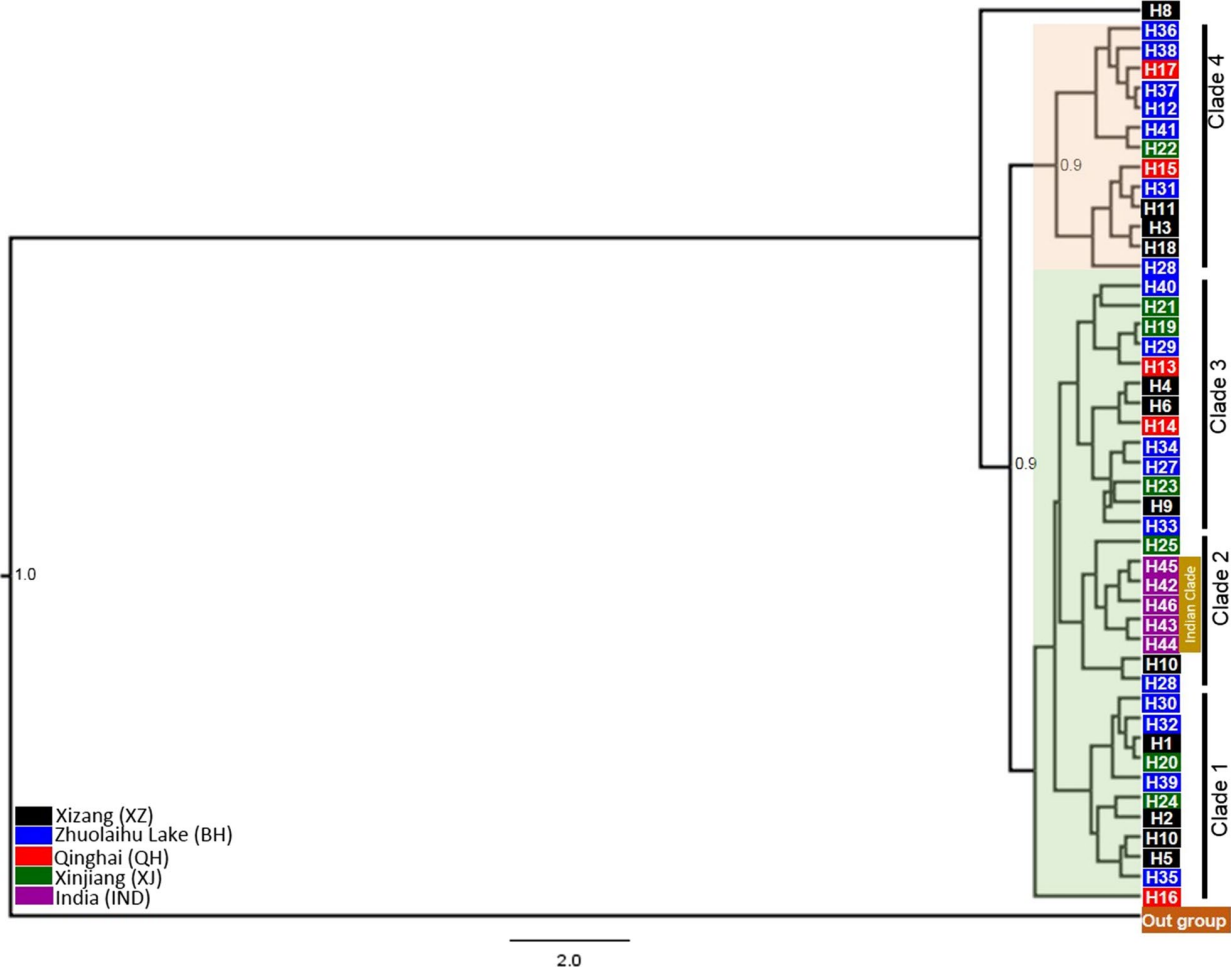
Bayesian phylogenetic relationships among Indian and Chinese populations of Tibetan antelope. All nodes with posterior probability >0.5 are displayed and sequence of *Capra hircus* (AF533441) was used as outgroup

The Bayesian phylogenetic tree, including samples from the Chinese populations, indicates the evolutionary relationship among the Indian and Chinese populations (Fig. 3). Broadly, all the H used in this study could be grouped into four clades. However, each clade except the clade 4 in the phylogenetic tree was poorly supported (<0.5 posterior probability), this may have been because of haplotype are shared between sampling locations on QTP so do not represent isolated populations. Zhang et al. [19] also reported low posterior probability for the majority of the clades except one having high posterior probability.

**Fig. 4:**
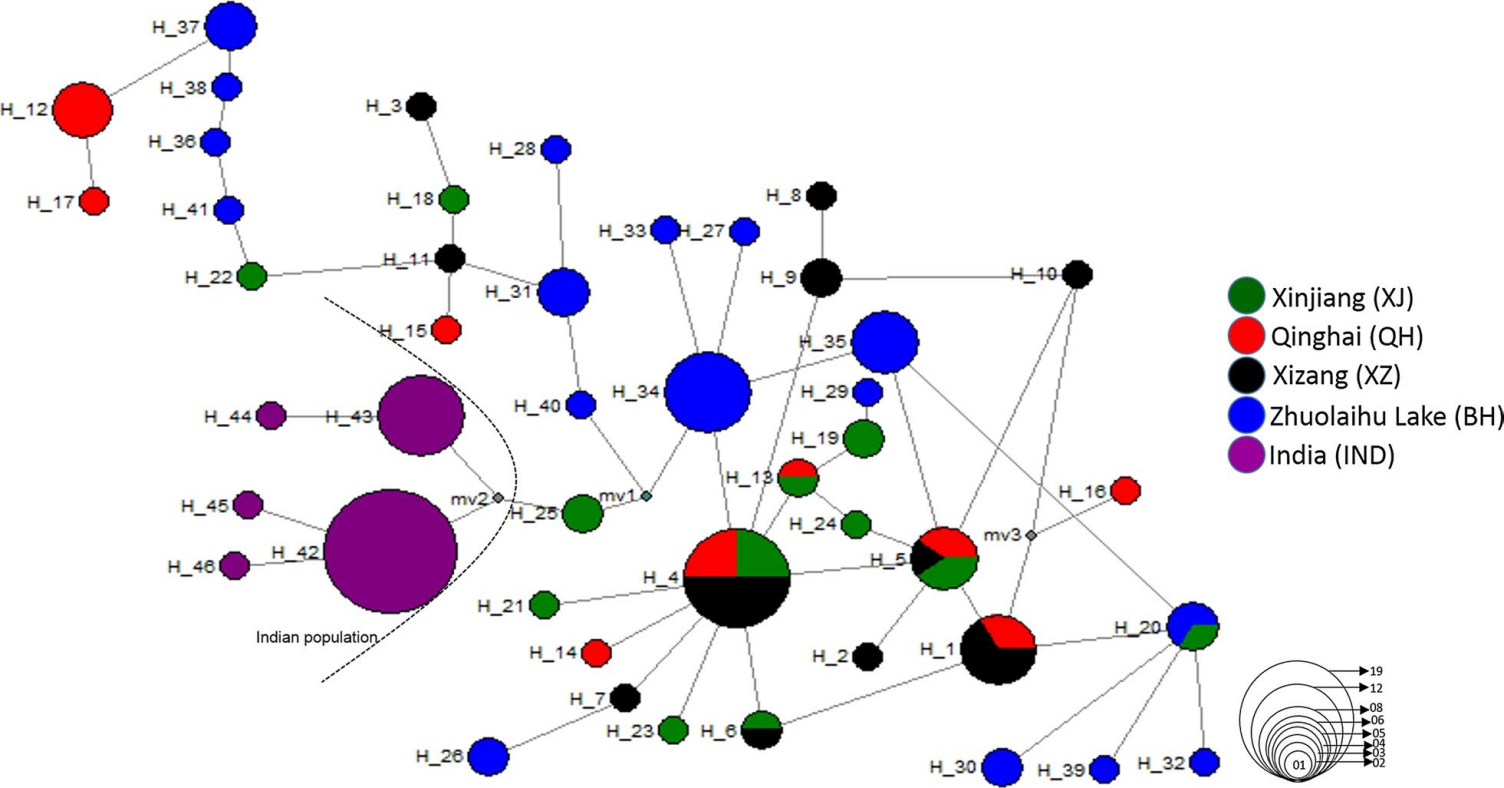
Median-joining networks showing genetic relationship among control region haplotypes of *Pantholops hodgsonii* (*n* = 118). Each *circle* represents a haplotype and its size is proportional to its total number of individual sequences. *Gray dots* indicate median vectors; *circle colour* denotes sampling location of different population

In addition, our median-joining (MJ) network (Fig. 4) analysis shows a clear pattern of haplotype distribution which spanned by a total of 46 H with two core haplotype (H4 and H42) and appears in star-like topology may corroborates a possible population expansion or the effect of ‘founder effect’. Haplotype 4 seems to be ancestral haplotype for all populations, whereas H42 was for the Indian population with a high frequency of samples covered. The Indian population is separated by one median vector from other Chinese populations, which is likely to be interpreted as missing haplotype that could have remained unsampled for the present study.

Demographic history analysis and star-like topology in the median-joining network analysis undertaken in the present study and other studies [19, 25] have clearly revealed historical expansion of the population over its range [25]; however, such analysis has rejected the hypothesis of Ruan et al. [10] that expansion in the population was at the end of last glacial maximum (LGM).

Presence of H in different clades as well as topology in MJ network analysis clearly suggest the absence of any geographic structure in the population of QTP [10, 19, 25], whereas the observed five H in the Indian population was not reported in previously studied populations. The distribution of mixed H among different populations of QTP (Fig. 3) clearly reveals the presence of high gene flow among these populations as observed by other authors [10, 19, 25]. The observed large scale migrations by females may have been the reason for the absence of unique haplotype in the four Chinese populations (XJ, XZ, QH, and BH) and may also indicate that no physical barriers are present along their migratory route on QTP. The absence of H of the western most population in studied populations of QTP so far indicate the lack of such long range movement, and this may be due to either the presence of any barrier (physical or anthropogenic) or other ecological reasons. Hence, this requires a detailed study to be undertaken for better understanding of environmental and ecological reasoning for such lack of movement between western most and QTP populations using other genetic markers or GPS collars. However, major clade II of the Indian population has H of Xizang, Zhuolaihu Lake, and Xinjiang. Interestingly, the haplotype of Xinjiang (H25) is basal to Indian population reveals that the Tibetan antelope population of Xinjiang might have migrated over Kunlun Mountains to the Aksi Chin and Ladakh, India. In addition, the presence of H25 (Xinjiang) and H28 (Zhuolaihu Lake) H with major clade of Indian populations (Fig. 3) also indicate the presence of small scale sporadic movements until the recent past over the Kunlun Mountains. Therefore, we believe that there should be gene flow between the Indian and Xinjiang Tibetan antelope population if there is no barrier present because geographical distance between Xinjiang and India is less than that between Xizang and Qinghai where large scale migration has been reported [10]. However, Sarkar et al. [11] has reported the seasonal migration in the Indian population during the early period of snowfall when animals migrate from Chang Chenmo Valley, India to the Tibet Autonomous Region, of China. More detailed studies are needed to document the extent of movement between these two areas. Arjin Shan National Nature Reserve (ASNNR) is the largest reserve in China and has a high density of Tibetan antelopes in the Kunlun Mountains. However, the migration of this population in the Xinjiang province was observed mainly towards the south and to some extent on the Aksai Chin plains, close to Xizang [5, 20]. The Kunlun Mountains are one of the longest mountain ranges in Asia, extending more than 2500 km [50] and are known to support permanent and migratory populations of Tibetan antelopes and other mammals [50, 51]. This could have been one of the reasons for the presence of the Xinjiang haplotype with the clade of the Indian population. However, the presence of H10 haplotype of Xizang with the clade of the Indian population may indicate the presence of other populations between Indian and Xizang. This is because individual females and their calves are known to migrate to other wintering habitats instead of their original habitats [20].

Schaller [5, 21] has recorded migration of the Tibetan antelope population of Xinjiang to the Aksai Chin plain close to Xizang and interchange of individuals between Xinjiang and Xizang populations near Tuzi Lake adjacent to the Chang Tang Reserve as well as close to HeiShi Beihu bordering Xinjiang. It is clear that there has been reported migration and exchange of individuals towards the western part in its range, but habitat suitability analysis is needed for a better understanding of the reasons for lack of major exchange of individuals between the western most (Depsang Plains close to DBO in northern Ladakh and Aksi Chin near Kunlun range) and other populations.

## Conclusions

Analysis of the mtDNA control region (259 bp) indicates the presence of relatively low genetic diversity in the surviving Tibetan antelope population in India in comparison to what has been reported for Tibetan antelope populations of QTP. Five H were observed in the Indian population, and so far, these have not been reported in previously studied populations on QTP. Bayesian phylogenetic tree analysis reveals the presence of four clades and indicates lack of population divergence in majority of populations. The absence of Indian H in other populations and a separate major clade in topology probably indicates the absence of female biased migration from India to QTP but requires studies to document the extent of migration present in these populations. The Chinese populations studied so far have a limited geographic structure, and correspondingly, the estimated gene flow among the four meta-populations (XJ, XZ, QH, and BH) indicates strong historical connections between the populations and making QTP as panmictic population. For effective conservation of the western-most population in India, our priority should be to plan genetic studies to include patterns of genetic diversity, relatedness, and population connectivity using mtDNA and nuclear markers of the reported populations around Ladakh and Aksai Chin to determine their relationship with other populations of QTP and identify if any barriers exist. This would enable us to understand and plan for effective conservation of Tibetan antelopes in the meta-population framework and plan adaptive management for restoring the genetic diversity of the eastern most populations. Besides, Tibetan antelopes will also require strong legislation because continued poaching for high wool demand may influence genetic diversity by reducing effective population size as well as population demography.

## Abbreviations

AKS: Aksai Chin
AAK: Anzara Anjum Khan
ASNNR: Arjin Shan national nature reserve
BH: breeding habitat
CCV: Chang Chenmo Valley
CITES: Convention On International Trade In Endangered Species
DBO: Daulat Beg Oldi
DNA: deoxyribonucleic acid
IND: India
KA: Khursheed Ahmad
mtDNA: mitochondrial DNA
MR: Mohamed Raza
PN: Parag Nigam
QH: Qinghai
QTP: Qinghai Tibetan Plateau
SPG: Surendra Prakash Goyal
VPK: Ved Prakash Kumar
XJ: Xinjiang
XZ: Xizang
